# Impact of changes in television viewing time and physical activity on longevity: a prospective cohort study

**DOI:** 10.1186/s12966-015-0315-0

**Published:** 2015-12-18

**Authors:** Sarah Kozey Keadle, Hannah Arem, Steven C. Moore, Joshua N. Sampson, Charles E. Matthews

**Affiliations:** Nutritional Epidemiology Branch, Division of Cancer Epidemiology and Genetics, National Cancer Institute, Bethesda, MD USA; Cancer Prevention Fellowship Program, Division of Cancer Prevention, National Cancer Institute, Bethesda, MD USA; Biostatistics Branch, Division of Cancer Epidemiology and Genetics, National Cancer Institute, Bethesda, MD USA

**Keywords:** Sedentary behavior, Television, Mortality, Prospective cohort

## Abstract

**Background:**

Television viewing is a highly prevalent sedentary behavior among older adults, yet the mortality risks associated with hours of daily viewing over many years and whether increasing or decreasing viewing time affects mortality is unclear. This study examined: 1) the long-term association between mortality and daily viewing time; 2) the influence of reducing and increasing in television viewing time on longevity and 3) combined effects of television viewing and moderate-to-vigorous physical activity (MVPA) on longevity.

**Methods:**

Participants included 165,087 adults in the NIH-AARP Diet and Health (aged 50–71 yrs) who completed questionnaires at two-time-points (Time 1: 1994–1996, and Time 2: 2004–2006) and were followed until death or December 31, 2011. Multivariable-adjusted Cox proportional hazards regression was used to estimate Hazard Ratios and 95 % confidence intervals (CI) with self-reported television viewing and MVPA and all-cause mortality.

**Results:**

Over 6.6 years of follow-up, there were 20,104 deaths. Compared to adults who watched < 3 h/day of television at both time points, mortality risk was 28 % greater (CI:1.21,1.34) those who watched 5+ h/day at both time-points. Decreasing television viewing from 5 + h/day to 3–4 h/d was associated with a 15 % reduction in mortality risk (CI:0.80, 0.91) and decreasing to <3 h/day resulted in an 12 % lower risk (CI:0.79, 0.97). Conversely, adults who increased their viewing time to 3–4 h/day had an 17 % greater mortality risk (CI:1.10, 1.24) and those who increased to 5+ h/day had a 45 % greater risk (CI:1.32, 1.58), compared to those who consistently watched <3 h/day. The lowest mortality risk was observed in those who were consistently active and watched < 3 h/day of television.

**Conclusions:**

We confirm that prolonged television viewing time was associated with greater mortality in older adults and demonstrate for the first time that individuals who reduced the amount of time they spent watching television had lower mortality. Our findings provide new evidence to support behavioral interventions that seek to reduce sedentary television viewing in favor of more physically active pursuits, preferably MVPA. Given the high prevalence of physical inactivity and prolonged television viewing in older adults, favorable changes in these two modifiable behaviors could have substantial public health impact.

**Trial registration:**

ClinicalTrials.gov number, NCT00340015

**Electronic supplementary material:**

The online version of this article (doi:10.1186/s12966-015-0315-0) contains supplementary material, which is available to authorized users.

## Background

The number of older adults (≥65 yrs) in the United States (US) is projected to increase from 40 million to 72 million over the next two decades [1], and individuals who reach age 65 are expected to live an additional 19 years [2]. Engaging in moderate-to-vigorous physical activity (MVPA) is an established component of healthy aging and initiating MVPA later in life can improve health and longevity [3–5]. However, older adults spend the majority of their waking day in sedentary behavior (i.e., sitting) [6]. Sedentary television viewing the most prevalent leisure-time sedentary behavior [7, 8], and on a given day nearly 90 % of older US adults watch television, for an average of 4.7 h per day [9]. Several studies have reported that television viewing is associated with increased risk of mortality, including 8 of the leading causes of death in the United States [10–17]. The discretionary nature, high prevalence and previously identified mortality risks from prolonged television viewing suggest reducing this behavior may have substantial public health impact, but key aspects of the television/mortality relationship remain poorly understood.

To date, prospective mortality studies have only measured television viewing at single point in time, and the magnitude of the risk estimates derived from these studies may be underestimated due to regression dilution bias [18]. Additionally, the impact of reducing or increasing time spent watching television and risk of mortality is not currently known. Such knowledge would help to estimate the effects of an intervention designed to decrease television viewing. Given the scarcity of randomized trials targeting television viewing and MVPA with mortality end-points, prospective studies with exposures measured at multiple time-points form a natural experiment that yield essential insights into the impact of changes in behavior on mortality risk. Accordingly, the purpose of this investigation, conducted within a large prospective study is to: 1) estimate the mortality risk associated with many years of prolonged television viewing, and 2) Evaluate the influence of changes in television viewing time on mortality risk. We also describe associations for MVPA over time and explore the independent and combined effects of changes in both television viewing and MVPA on longevity.

## Methods

### Cohort information and informed consent

The NIH-AARP Diet and Health Study is a prospective cohort that was established in 1995–96 when 566,398 AARP members (aged 50–71 years) who lived in one of six states (California, Florida, Louisiana, New Jersey, North Carolina, and Pennsylvania) or two metropolitan areas (Atlanta, Georgia, and Detroit, Michigan) responded to a questionnaire about their medical history, diet, and demographics (ClinicalTrials.gov number, NCT00340015) [19]. Within six months, those without colon, breast or prostate cancer were mailed a Risk Factor Questionnaire that asked about sedentary behaviors and physical activity (*n* = 334,908). In 2004–2006, all participants were mailed a Follow-up Questionnaire that asked detailed questions about active and sedentary behaviors, and other risk factors (*n* = 313,835). Completion of the questionnaires was considered to imply informed consent. The Special Studies Institutional Review Board of the US National Cancer Institute approved the study.

### Analytic study design and End point ascertainment

**Fig. 1:**
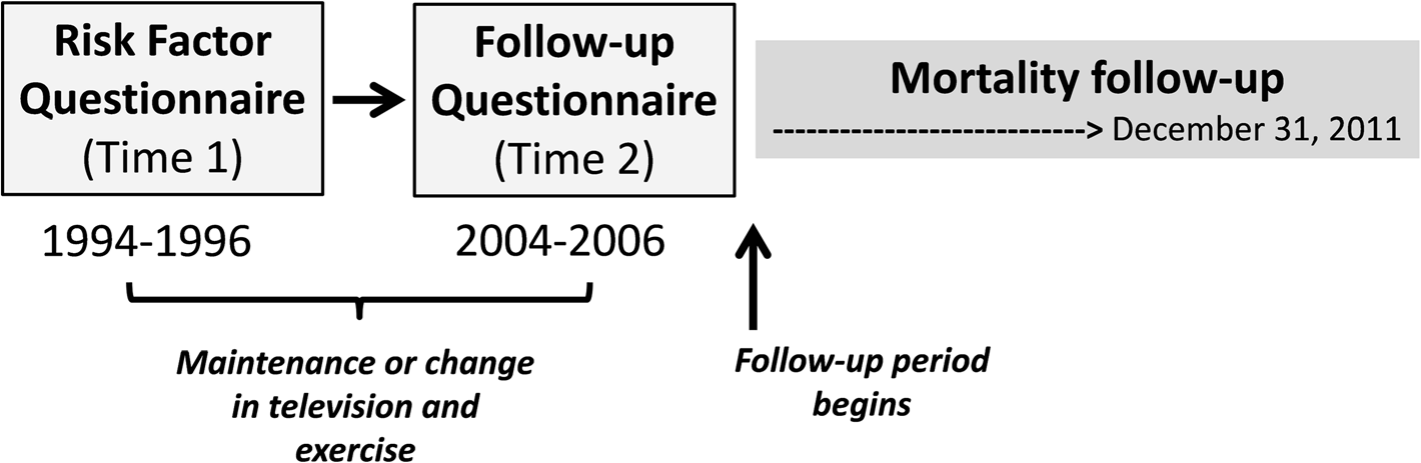
Study Design

There were 221,189 individuals who returned both the Risk Factor (Time 1) and Follow-up Questionnaire (Time 2) making them eligible for this analysis (Fig. 1). Of these, we excluded proxy respondents (*n* = 15,602) and those missing physical activity and television data (*n* = 22,785). To minimize reverse causality we also excluded those who had poor self-reported health at Time 1 or Time 2 (*n* = 5137) or who had missing data for this variable (*n* = 12,137). The final sample included 165,087 individuals. Cohort members were followed by linkage to the US Postal Service National Change of Address database, through processing of undeliverable mail, address change services, and direct contact. Vital status was determined through linkage with the Social Security Administration Death Master File [20], and the National Death Index [21]. The primary end point was all-cause mortality. Verification of vital status was available for >95 % of the cohort.

### Assessment of television viewing and MVPA

A detailed description of our estimates of television viewing and MVPA are provided in our supplementary materials (Additional file 1). Briefly, we harmonized the self-reported television and MVPA responses into the most meaningful categories allowed by both measures and that ensured adequate cell sizes for change analyses. The categories were <3, 3–4, and 5+ hours per day (h/day) for television viewing and <1, 1–4, and 4+ hours per week (h/wk) for MVPA participation.

Studies reporting the reliability of television viewing questionnaires indicate moderate to high reliability (ICC = 0.78–0.93) [22–24] and are similar in general adults, unemployed and retired individuals (ICC = 0.76–0.93) [25]. Gardiner and colleagues showed reported sedentary behaviors were as responsive to change as the ActiGraph 100 count cut-point method to estimate sedentary time [22]. Criterion validity of these particular television viewing questions has not been evaluated, but the questions are similar to questions that have acceptable validity compared to behavioral logs (r^2^ = 0.61) [26] and an electronic TV monitor (r^2^ = 0.51) [27]. The MVPA questions used at Time 2 are nearly identical to questions that have been validated against physical activity diaries, R = 0.62 and 0.65, in the Harvard Cohorts [28, 29].

### Covariates

To identify potential confounders we examined each of the self-reported factors listed in Table 1 in age (yrs) and gender (male or female), adjusted models of change in TV time and mortality and retained only those demonstrating evidence of confounding (>5 % change). These factors were education (<12 yrs, high school graduate, some college, college graduate, or missing), smoking history (never; quit, <20 cigarettes/d; quit, >20 cigarettes/d; current, <20 cigarettes/d; current, 20 cigarettes/d; or unknown), history of heart disease (yes/no), other chronic conditions (yes/no), health status (fair, good, very good or excellent), and change in BMI categories between Time 1 and Time 2 (<25, 25–30, >30 kg/m^2^). Extreme BMI values (i.e., <15 or >60 kg/m^2,^) were set as missing. Due to know differences in levels of these exposures by television viewing patterns, we also adjusted for race (white, black, other, or missing) and depression (self-reported physician diagnosed depression [yes, no, or missing]). Missing values were included as covariates and were less than 8 % for all included variables. Mutual adjustment for MVPA and TV viewing duration was also completed, as necessary. For covariates that could have changed between Time 1 and Time 2 we used the values reported at Time 2.

**Table 1:** Participant characteristics by television viewing and MVPA participation at Time 2*: the NIH-AARP Diet and Health Study

	Television viewing (h/day)			MVPA (h/wk)		
	<3	3–4	5+	<1	1–4	4+
N	70963	59162	35062	38058	53237	72951
Age (y)	70.1 (5.4)	71.0 (5.2)	71.3 (5.1)	70.7 (5.4)	70.8 (5.3)	70.6 (5.2)
BMI (kg/m^2^)	26.1 (4.2)	27.0 (4.5)	28.0 (5.2)	28.0 (5.3)	26.9 (4.6)	26.1 (4.0)
Education-some college (%)	74.8	65.1	57.4	58.8	65.9	73.6
Married (%)	71.2	69.9	63.7	64.5	66.9	73.1
Race (% white)	94.1	93.9	92.0	92.5	93.5	94.2
Very good/excellent health (%)	60.8	49.3	39.7	38.4	48.2	62.3
Retired (%)	70.5	81.8	86.9	75.1	76.8	80.5
Sleep <7 h/night (%)	26.9	27.9	29.1	31.3	28.0	25.7
MVPA 4+ h/wk (%)	46.2	35.5	18.3	-	-	-
Television 5+ h/day (%)	-	-	-	27.4	20.8	18.3
Ever diagnosed with (%):						
Coronary heart disease	18.1	22.7	25.6	22.4	21.2	21.0
Cancer	12.8	13.6	14.1	13.7	13.3	13.3
Chronic condition	6.0	8.1	11.2	11.1	8.2	6.0
Degenerative disease	0.7	1.0	1.2	1.3	1.0	0.7
Depression	11.6	12.2	15.4	16.1	13.7	10.1
Hypertension	48.8	55.7	60.5	57.7	55.5	50.4
High Cholesterol	52.0	57.5	59.6	55.4	56.3	55.2
Diabetes	11.3	15.5	20.3	18.6	15.5	12.1

Information at Time 2 was reported on the Follow-up Questionnaire, administered 2004–2006. Values in parentheses are standard deviation. Degenerative diseases refer to Parkinson’s Diease, Amyotrophic lateral sclerosis or Multiple Sclerosis

### Statistical analyses

Cox proportional hazards regression models were used to obtain adjusted hazard ratios (HR) and 95 % confidence intervals (CI). Follow-up time was calculated from the Time 2 questionnaire until death or December 31, 2011 (Fig. 1). First we estimated the HR and 95 % CI for television viewing at each time point. To evaluate the long-term risks associated with television viewing we then estimated HRs for those who reported maintaining the same amount of television viewing at Time 1 and 2 (i.e., no change). To estimate associations for increasing or decreasing television viewing, we fit models stratified by television viewing at Time 1, using those who maintained the same television viewing time at both time-points as the reference group. Parallel analyses were completed for MVPA participation. Sensitivity analyses were conducted for our main analysis to evaluate potential reverse causality by excluding the first year of follow up and excluding individuals with cardiovascular disease, cancer, renal disease or neurodegenerative conditions (i.e., Parkinson’s Disease, Amyotrophic lateral sclerosis or Multiple Sclerosis). We also compared the HR’s for change in television and MVPA in men and women separately, by BMI status (<25 kg/m^2^ vs. ≥ 25 kg/m^2^) and in younger and older individuals based on median age at Time 2.

We examined the joint effects of changes in both television and MVPA. To preserve power for this analysis we evaluated only two categories for television viewing (<3 and 3 + h/day) and MVPA participation (<1 and 1 + h/wk) and set the highest risk group (i.e., those reporting high levels of television (3+ h/day) and low levels of MVPA (<1 h/wk) at both time-points) as referent. Spearman correlations were assessed for our measures of television and MVPA over time.

We tested for violations of the proportional hazards assumption in our primary analyses by comparing, using the likelihood ratio test, models with and without the log (time) by exposure group interactions [30]. Given evidence of minor violations for some of the models tested, we pursued secondary analyses where we allowed the effect of each exposure category to change at follow-up time of three years. The risk estimates for those who died within the first 3 years of follow-up (*N* = 6587) were slightly stronger than deaths occurring after 3 yrs (13,517) (Additional file 1: Tables S1–S2). However, the direction the associations for all of the models were consistent over time and did not warrant further stratification of results by follow-up time. SAS 9.3 was used for all analysis and statistical significance was set at *P* < 0.05.

## Results

Over 6.6 years of follow-up, there were 20,104 deaths. Participant characteristics at Time 2 are presented in Table 1. Those who watched more television and engaged in less MVPA tended to be less educated, less likely to report excellent or very good health and more likely to be retired and have received diagnoses of hypertension or depression. Those who watched more television also had higher rates of coronary heart disease, diabetes and high cholesterol. Additional file 1: Tables S3–S4 show the participant characteristics across the 9-change categories for television viewing and MVPA, respectively.

First we evaluated the risks associated with television viewing and MVPA in separate models for Times 1 and 2 (Table 2). Mortality was greater for prolonged television viewing at both time points after adjusting for demographic factors, smoking, body mass index, self-reported health, pre-existing disease and MVPA participation (Table 2). The mortality risk increased at both Time 1 (HR [95 % CI]) (1.11 [1.06, 1.15]) and Time 2 (1.29 [1.25, 1.34]) for those who watched 5+ vs. < 3 h/day. Engaging in MVPA (<1 h/wk vs >4 h/wk) was associated with decreased mortality risk at both time-points (Time 1: 0.98 [0.86–0.92] and Time 2: 0.69 [0.66–0.71]) (Table 2). The Spearman correlations television viewing and MVPA participation between Time 1 and Time 2 were R = 0.53 and R = 0.31, respectively. In the Time 2 questions, we examined the proportion of MVPA that came from different types of activity and found most of the reported MVPA came from walking (54 %) and 12 % was reported as vigorous activity.

**Table 2:** Associations (HR [95 % CI]) between television viewing and MVPA participation and mortality at Time 1 and Time 2, the NIH-AARP Diet and Health Study

	Time 1 (1996–1997)				Time 2 (2004–2006)			
Television viewing								
	<3 h/day	3–4 h/day	5 + h/day	p-value	<3 h/day	3–4 h/day	5 + h/day	p-value
N (deaths)	56385 (6061)	63278 (9191)	25420 (4852)		64275 (6688)	51615 (7547)	29193 (5869)	
Age and sex	1.0 (Referent)	1.21 (1.17, 1.25)	1.53 (1.47, 1.59)	<0.001	1.0 (Referent)	1.28 (1.24, 1.32)	1.71 (1.65, 1.77)	<0.001
Fully-adjusted model	1.0 (Referent)	1.04 (1.01, 1.08)	1.11 (1.06, 1.15)	<0.001	1.0 (Referent)	1.11 (1.08, 1.15)	1.29 (1.25, 1.34)	<0.001
Moderate-to-vigorous physical activity								
	<1 h/wk	1–4 h/wk	>4 h/wk		<1 h/wk	1–4 h/wk	>4 h/wk	
N (deaths)	32182 (6061)	37208 (5058)	75693 (9543)		31622 (6436)	47430 (6748)	66031 (6920)	
Age and sex	1.0 (Referent)	0.80 (0.77, 0.83)	0.70 (0.67, 0.72)	<0.001	1.0 (Referent)	0.70 (0.68, 0.72)	0.51 (0.49, 0.53)	<0.001
Fully-adjusted model	1.0 (Referent)	0.92 (0.89, 0.96)	0.89 (0.86, 0.92)	<0.001	1.0 (Referent)	0.82 (0.79, 0.85)	0.69 (0.66, 0.71)	<0.001

Fully adjusted model: age (yrs), sex (male or female), race (white, black, other, or missing), education (<12 yrs, high school graduate, some college, college graduate, or missing), smoking history (never; quit, <20 cigarettes/d; quit, >20 cigarettes/d; current, <20 cigarettes/d; current, .20 cigarettes/d; or unknown), history of heart disease (yes/no), depression( yes/no, missing), other chronic conditions (yes/no), health status (fair, good, very good or excellent). and change in BMI category from Time 1 to time 2 (<25, 25– < 30, >30, or missing). Television viewing models were adjusted for MVPA (<1, 1–4, >4 h/wk) and MVPA models were adjusted for television (<3, 3–4, 5+ h/day). Time 1 was 1995–1996, Time 2 is 2004-2005

**Fig. 2:**
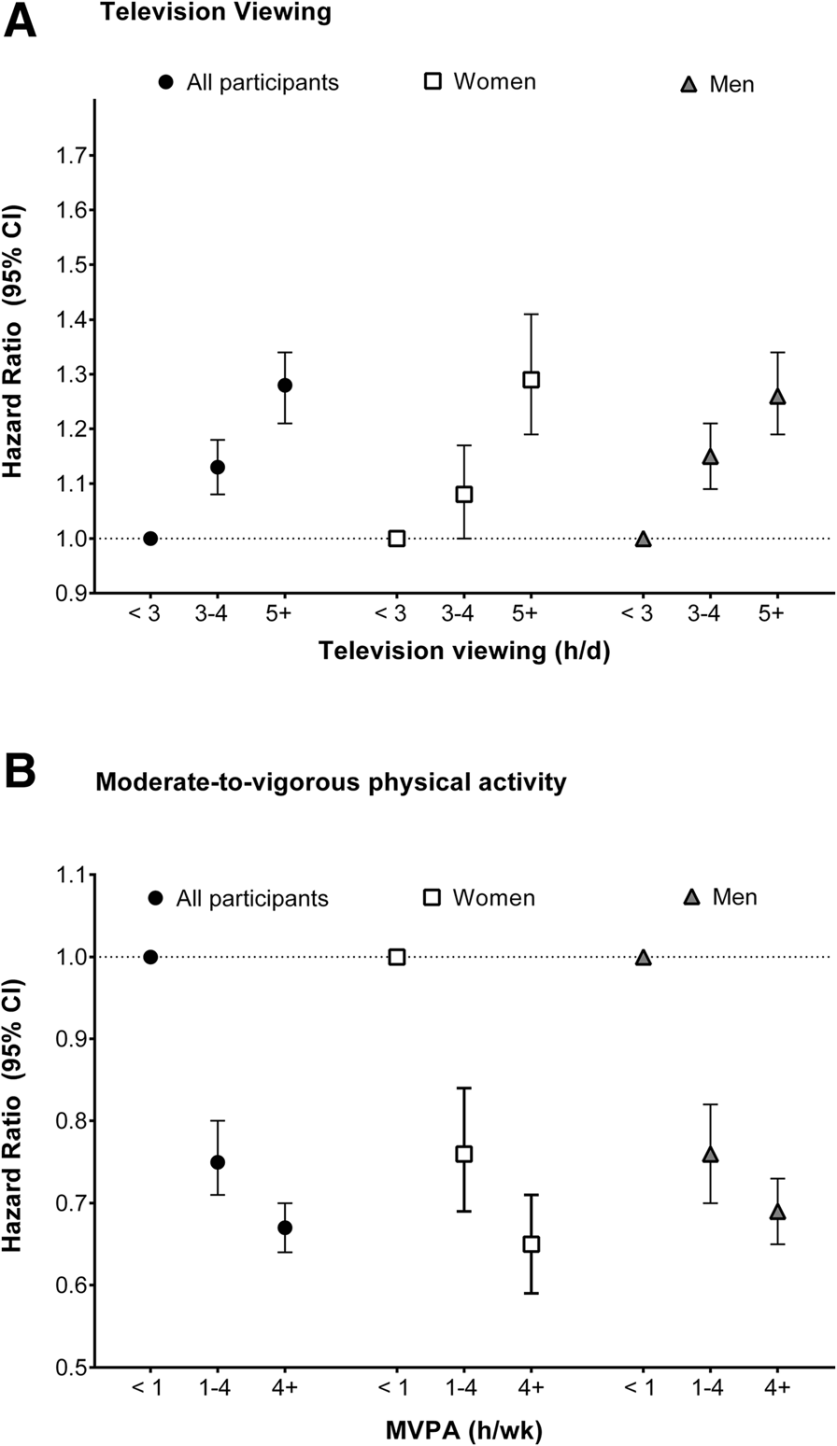
Associations between long-term patterns of television viewing (Panel **a**) and MVPA participation (Panel **b**) on mortality, in all participants and by sex, the NIH-AARP Diet and Health Study. * Participants in this analysis reported the same category of television viewing or MVPA at both Time 1 (1995–1996) and Time 2 (2004–2006). Fully adjusted model: age (yrs), sex (male or female), race (white, black, other, or missing), education (<12 yrs, high school graduate, some college, college graduate, or missing), smoking history (never; quit, <20 cigarettes/d; quit, >20 cigarettes/d; current, <20 cigarettes/d; current, .20 cigarettes/d; or unknown), history of heart disease (yes/no), other chronic conditions (yes/no), depression (yes/no, missing), health status (fair, good, very good or excellent), and change in BMI category from Time 1 to Time 2 (<25, 25– < 30, >30, or missing), and MVPA or television viewing respectively

To estimate the mortality risk associated with many years of prolonged television viewing and MVPA participation, we evaluated those who maintained the same level of each behavior at both time-points (Fig. 2, Panel a). Compared to those who watched <3 h/day at both time-points, mortality risk was higher for individuals who consistently watched 3–4 h/day (1.13 [1.08, 1.18]) and for those who consistently watched 5+ h/day (1.28 [1.22, 1.35]). Those who maintained >4 h/wk of MVPA over time were at a 33 % lower risk (0.67 [0.64, 0.70]) compared to those reporting <1 h/wk of MVPA at both time points (Fig. 2, Panel b). Results for both television viewing and MVPA were similar for women and men.

### Changes in television viewing and MVPA participation

**Fig. 3:**
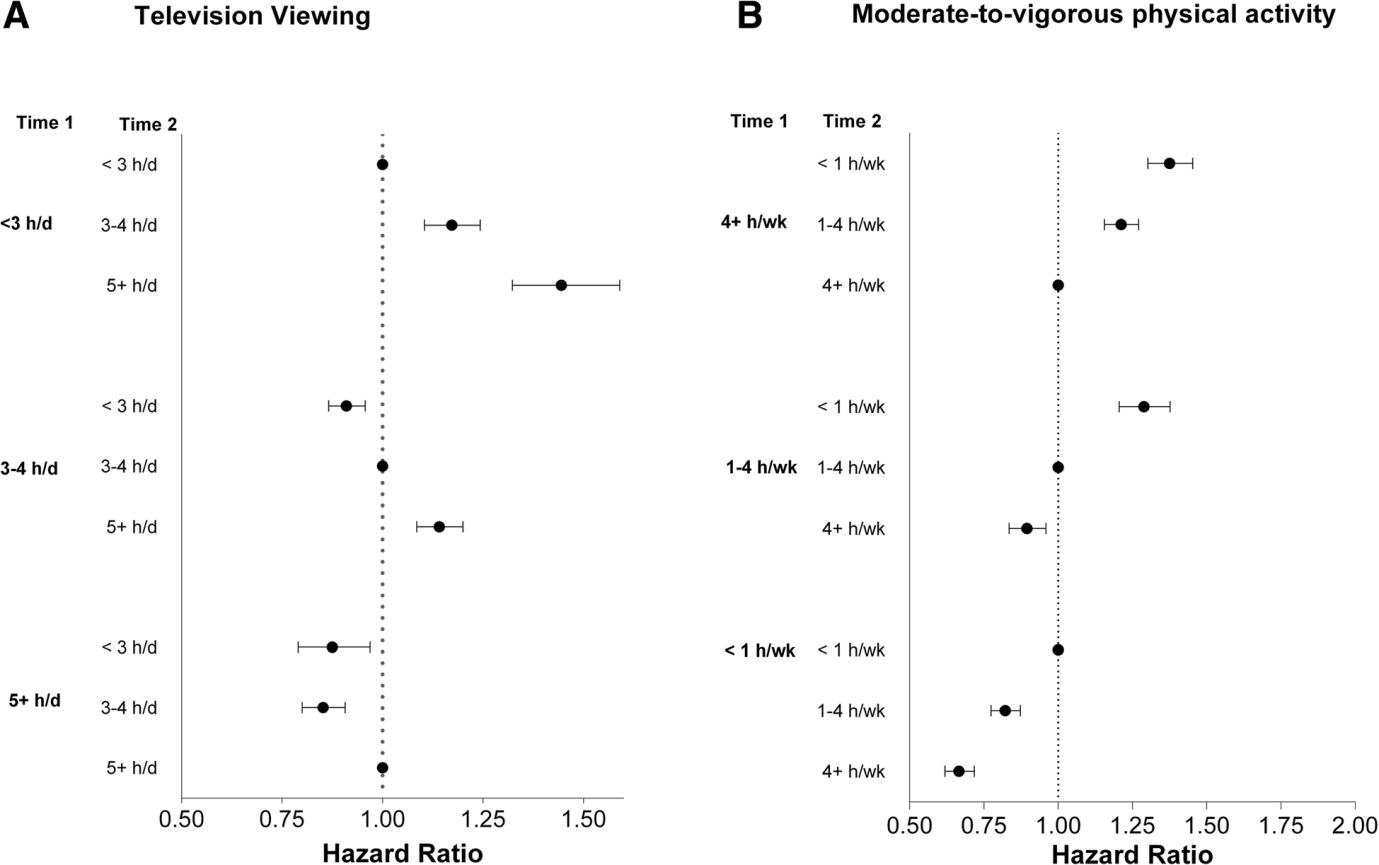
Associations between changes in television viewing (Panel **a**) and Moderate-to-vigorous physical activity (Panel **b**) over time on mortality, the NIH-AARP Diet and Health Study. *Fully adjusted model: age (yrs), sex (male or female), race (white, black, other, or missing), education (<12 yrs, high school graduate, some college, college graduate, or missing), smoking history (never; quit, <20 cigarettes/d; quit, >20 cigarettes/d; current, <20 cigarettes/d; current, .20 cigarettes/d; or unknown), history of heart disease (yes/no), other chronic conditions (yes/no), depression (yes/no), health status (fair, good, very good or excellent), depression (yes/no, missing) and change in BMI category from Time 1 to time 2 (<25, 25– < 30, >30, or missing) MVPA or television viewing respectively

Among those with higher levels of television viewing at baseline, individuals who reduced their amount of television viewing over time had significantly lower mortality compared to those who maintained the same level of viewing (Fig. 3, Panel a). Mortality risk was 15 % lower in adults who reduced their viewing from 5+ h/day at Time 1 to 3–4 h/day at Time 2 (0.85 [0.80, 0.91]), and 12 % lower for those who reduced to <3 h/day at Time 2 (0.88 [0.79, 0.97]), compared to those who consistently watched 5+ h/day. Conversely, those who increased television viewing at Time 1 from < 3 h/day to3-4 h/day had an increased risk (1.17 [1.10, 1.24]) and those who increased to 5+ h/day had a greater risk (1.45 [1.32, 1.58]), compared to those who consistently watched <3 h/day.

Reduction in MVPA was also associated with higher mortality and increased MVPA was associated with lower mortality (Fig. 3, Panel b). For example, mortality risk was lower among individuals who increased their reported MVPA from < 1 h/wk at Time 1 to 1–4 h/wk (0.82 [0.77, 0.87]) and those who increased MVPA from < 1 h/wk to 4+ h/day (0.67 [0.62, 0.72]), compared to those who reported <1 h/wk of MVPA over time.

Results of the sensitivity analyses excluding the first year of follow-up and those with chronic conditions were similar to the overall values suggesting these factors did not exert a major impact on our results and minimizing reverse causation concerns (Table 3, Additional file 1: Table S5–S6). For example, for participants who decreased television viewing from 5 + h/day to 3–4 h/day, the overall estimate was 0.85 (0.80, 0.91) and once those with any history chronic conditions were excluded the estimate was 0.84 (0.76–0.93). Similarly, the estimates for increasing from 3 + h/day to 4 + h/day were 1.45 (1.32, 1.58) for the overall sample and 1.41 (1.22, 1.62) for those without any chronic conditions. Risk estimates were also similar in normal weight compared to overweight/obese participants, and men and women (Table 3, Additional file 1: Table S6). The risks of television viewing appeared to be slightly stronger in younger individuals, indicated by a significant age group by television interaction (*P* < 0.05).

**Table 3:** Sensitivity Analyses for association between changes in television viewing and mortality: the NIH-AARP Diet and Health Study

Time 1		<3 h/day			3–4 h/day			5+ h/day		
Time 2	No. deaths	<3 h/d	3–4 h/d	5 + h/d	<3 h/d	3–4 h/d	5 + h/d	<3 h/d	3–4 h/d	5 + h/d
Overall	20,104	1.0 (referent)	1.17 (1.10,1.24)	1.45 (1.32,1.58)	0.91 (0.87,0.96)	1.0 (referent)	1.14 (1.09,1.20)	0.88 (0.79,0.97)	0.85 (0.80,0.91)	1.0 (referent)
Men	13,251	1.0 (referent)	1.21 (1.13,1.30)	1.39 (1.25,1.56)	0.94 (0.89,1.00)	1.0 (referent)	1.11 (1.04,1.18)	0.92 (0.81,1.05)	0.86 (0.80,0.94)	1.0 (referent)
Women	6853	1.0 (referent)	1.09 (0.98,1.21)	1.54 (1.32,1.79)	0.84 (0.77,0.92)	1.0 (referent)	1.21 (1.11,1.32)	0.82 (0.69,0.97)	0.84 (0.76,0.92)	1.0 (referent)
Normal weight	8560	1.0 (referent)	1.16 (1.06,1.27)	1.43 (1.25,1.64)	0.87 (0.81,0.94)	1.0 (referent)	1.17 (1.08,1.26)	0.86 (0.74,1.01)	0.86 (0.77,0.95)	1.0 (referent)
Overweight/obese	11,544	1.0 (referent)	1.18 (1.09,1.28)	1.46 (1.30,1.64)	0.94 (0.88,1.01)	1.0 (referent)	1.13 (1.06,1.20)	0.89 (0.78,1.02)	0.85 (0.78,0.92)	1.0 (referent)
Younger	5968	1.0 (referent)	1.14 (1.03,1.26)	1.56 (1.36,1.80)	0.92 (0.84,1.01)	1.0 (referent)	1.16 (1.06,1.28)	0.79 (0.65,0.96)	0.75 (0.66,0.84)	1.0 (referent)
Older	8231	1.0 (referent)	1.19 (1.11,1.28)	1.36 (1.21,1.53)	0.91 (0.85,0.96)	1.0 (referent)	1.13 (1.07,1.20)	0.91 (0.81,1.03)	0.89 (0.83,0.96)	1.0 (referent)
>1 yr Follow-up	18,506	1.0 (referent)	1.17 (1.10,1.24)	1.44 (1.31,1.58)	0.91 (0.86,0.95)	1.0 (referent)	1.12 (1.06,1.18)	0.87 (0.78,0.97)	0.85 (0.80,0.91)	1.0 (referent)
Excluding chronic conditions	8231	1.0 (referent)	1.11 (1.01,1.21)	1.41 (1.22,1.62)	0.94 (0.87,1.01)	1.0 (referent)	1.12 (1.03,1.21)	0.88 (0.75,1.02)	0.84 (0.76,0.93)	1.0 (referent)

*Note*: Time 1 was 1995–1996, Time 2 was 2004–2005. Chronic conditions included reported history of cardiovascular disease, cancer, renal disease or neurodegenerative conditions (Parkinson’s Diease, Amyotrophic lateral sclerosis or Multiple Sclerosis). Younger individuals were below median age (71.2y at Time 2) and older were greater than or equal to the median age. Nomal-weight was BMI <25 kg/m^2^ and overweight/obese was greater than or equal to 25 kg/m^2^

Fully adjusted model: age (yrs), sex (male or female), race (white, black, other, or missing), education (<12 yrs, high school graduate, some college, college graduate, or missing), smoking history (never; quit, <20 cigarettes/d; quit, >20 cigarettes/d; current, <20 cigarettes/d; current, .20 cigarettes/d; or unknown), history of heart disease (yes/no, missing), other chronic conditions (yes/no , missing), depression (yes/no, missing), health status (fair, good, very good or excellent), and change in BMI category from Time 1 to Time 2 (<25, 25– < 30, >30, or missing) and MVPA

**Table 4:** Combined effects of maintenance and changes in both television viewing and MVPA between Time 1 and Time 2, the NIH-AARP Diet and Health Study

			Television viewing		
MVPA		Maintain high (≥3 h/day)	Increased (<3 to ≥3 h/day)	Reduced (≥3 to <3 h/day)	Maintain low (<3 h/day)
Maintain low (<1 h/wk)	No. Deaths Hazard ratio (CI)	1700 1.0 (referent)	251 1.03 (0.90, 1.17)	330 0.91 (0.81, 1.02)	334 0.80 (0.72, 0.90)
Reduced (≥1 to <1 h/wk)	No. Deaths Hazard ratio (CI)	2257 0.91 (0.85, 0.97)	428 1.11 (1.0, 1.24)	502 0.79 (0.71, 0.87)	634 0.83 (0.76, 0.91)
Increased (<1 to ≥1 h/wk)	No. Deaths Hazard ratio (CI)	1684 0.77 (0.72, 0.82)	283 0.69 (0.61, 0.78)	400 0.71 (0.63, 0.79)	521 0.66 (0.60, 0.73)
Maintain high ( ≥1 h/wk)	No. Deaths Hazard ratio (CI)	5590 0.72 (0.68, 0.76)	1223 0.75 (0.69, 0.81)	1580 0.62 (0.58, 0.67)	2387 0.60 (0.56, 0.64)

Time 1 (1995–1996), Time 2 (2004–2006). MVPA (Moderate-to-vigorous physical activity) Fully adjusted model: age (yrs), sex (male or female), race (white, black, other, or missing), education (<12 yrs, high school graduate, some college, college graduate, or missing), smoking history (never; quit, <20 cigarettes/d; quit, >20 cigarettes/d; current, <20 cigarettes/d; current, .20 cigarettes/d; or unknown), history of heart disease (yes/no), depression (yes/no, missing), other chronic conditions (yes/no), health status (fair, good, very good or excellent), and change in BMI category from Time 1 to Time 2 (<25, 25– < 30, >30, or missing)

### Combined effects of maintenance and changes in television viewing and MVPA participation

To estimate the risk associated with various combinations of maintenance and change in television viewing time and MVPA participation we compared each behavioral category to a high risk referent group (i.e., those reporting high levels of television (3+ h/day) and low levels of MVPA (<1 h/wk) at both time-points); Table 4). Relative to the high-risk referent group, individuals who were active and watched little television at both time-points had the lowest mortality risk (0.60 [0.56–0.64]). Compared to those who did low MVPA and watched high amounts of television, individuals who maintained low levels of MVPA but watched low levels of television at both time-points had a 20 % reduction in risk (0.80 [0.72, 0.90]), while those who maintained high levels of television but consistently engaged in MVPA had a 28 % lower mortality risk (0.72 [0.68, 0.76]). Those who made positive changes in both behaviors (i.e., increased MVPA and decreased television viewing over time) had a 29 % lower mortality risk (0.71 [0.63, 0.79]), compared to those who engaged in little MVPA and watched more television at both time-points.

## Discussion

In this large prospective study of older adults we confirm and extend previous research by quantifying the adverse impact on mortality for higher levels of television viewing over many years. We also report, for the first time, that older adults who increased their amount of viewing over time had higher mortality, and that individuals who chose to reduce the amount of time they spent watching television had significantly lower mortality. While previous studies have demonstrated that older adults who increase participation in MVPA have lower mortality [3, 4, 31], until now it was unknown whether reductions in television viewing might also confer mortality benefits. Our findings suggest that reducing time spent in the most prevalent leisure time sedentary behavior in the US (television viewing) can improve health and longevity. Mortality benefits for increasing MVPA were robust and the greatest benefit was observed for those who watched little television and engaged in MVPA at least 1 h/wk. Given the high prevalence of physical inactivity and prolonged television viewing among older adults in the US and other western countries, favorable changes in these two modifiable behaviors could have a substantial clinical and public health impact.

Prior studies of television viewing and mortality evaluated excess risk at a single point in time [10–16] and these studies may have underestimated the true strength of this association due to regression dilution bias, or the failure to account for changes in behavior over time [17]. However, the risk estimate we observed for prolonged television viewing over many years, which relied on two measures of television viewing about 10 years apart, were largely consistent with the strength of association reported in earlier studies. A 2011 meta-analysis estimated a 13 % increase in mortality risk per 2-h of television viewing [13], while a 2015 meta-analysis showed a 22 % increased mortality risk in the highest vs lowest category of sedentary time (including television), with 12/14 prospective studies reporting positive associations [32]. In the present study, individuals who consistently watched television for 5+ h/day over many years had a 28 % higher mortality risk than those who consistently watched <3 h/day. This result has significant implications because the majority of older adults in the US watch television on a given day, and the average viewing time is nearly 5 h/day, indicating that a large proportion of older adults may be at increased risk due to prolonged television viewing.

We also demonstrated that adults who *increased* their viewing over time had a 17–45 % higher risk, and that individuals who chose to *reduce* their viewing time had an 12–15 % lower mortality, compared to those who maintained the same viewing patterns over time. In the only mortality studies of which we are aware to examine changes in sedentary behavior, Leon-Munoz reported that reductions in reported sitting time from 5+ to < 5 h/day over two years was associated with a non-significant 14 % lower mortality risk (0.86 [0.70–1.05]), compared to consistently sitting <5 h/day [33] and Lee et al., reported that women who reduced sitting had a 29 % lower mortality risk compared to those who maintained high levels of sitting [34] . Collectively, these data supports the idea that reducing sedentary behavior may have important mortality benefits and suggests that intervention strategies to reduce prolonged television viewing time could be an important behavioral approach to improve the health and longevity of older adults.

There are plausible behavioral and biological mechanisms that explain our findings. Television viewing is adversely associated with weight gain [35, 36], diabetes [13], glucose homeostasis [37], and other cardiometabolic risk factors [38]. In addition to the aforementioned metabolic risk factors, reductions in television viewing may also increase overall physical activity. A randomized trial found that a 2.9 h/day reduction in television viewing resulted in an increase in objectively measured physical activity of about 120 kcal/day [39], which is roughly equivalent to walking one mile per day. Prolonged television viewing also has been associated with lower cardiorespiratory fitness [40] and two weeks of imposed inactivity has been found to reduce cardiorespiratory fitness even in healthy young adults [41]. Although such data are unavailable for older adults, we hypothesize that the mortality benefits associated with reduced television time may be mediated by increased physical activity [43] and possibly greater cardiorespiratory fitness, a powerful determinant of mortality [42]. Our hypothesis is that sedentary television viewing is replaced with physical activity, which provides the mortality benefits observed for those who reported reducing television viewing. Future studies are needed to understand more completely the mechanisms underlying these associations, and to define the type and intensity of physical activity that may be providing the hypothesized replacement benefit. To fully understand the mortality associations between the different components of activity (i.e., sedentary, light, moderate) better measurements, such as previous day recalls [44] or activity monitors that capture total daily activity are needed in prospective studies. Future intervention studies that include robust measures are needed to determine how to effectively reduce sedentary television viewing, increase physical activity and determine the subsequent impact on health-related outcomes.

Strengths of our report include a study design that defined the temporal sequence between changes in behavior and mortality in a large cohort enabling precise risk estimates. We also took a number of steps to address the potential for reverse causation. First, the change analysis alleviates some concerns, as this potential bias is expected to be minimal when an individual who watches a lot of television at baseline subsequently reduces their television viewing, or when an inactive individual increases MVPA. We conducted a number of sensitivity analyses by different follow-up times and excluding those reporting any chronic disease (e.g., heart disease, cancer, renal disease, and degenerative neurological conditions) and our results remained consistent (Table 3, Additional file 1: Table S5–S6). In addition, we adjusted for important potential confounders including age, demographics, smoking, BMI, and reported health status and considered many others (e.g., sleep duration and diabetes) that did not change our results. It is important to note that the change results were adjusted for change in BMI and health status, both of which could be on the causal pathway between exposure and outcome, but also could be confounders thus we elected to provide a conservative risk estimate. Due to concerns about depression causing increases in television viewing, we adjusted for depression as well and the effect estimates were virtually identical.

The limitations of our study must also be considered. Television viewing has been associated with an unhealthy diet and while we could not control for recent diet in this study, we previously reported that diet quality was not a confounder of the television mortality association [10, 17]. We relied on self-selected maintenance or changes in behavior over time, rather than an experimental manipulation *per se*, therefore unmeasured residual confounding associated with these changes could have influenced our results. We do not know the types of activity that replaced sedentary television viewing for those who reported reducing this behavior. The measures of television and MVPA were both self-reported and the specific questionnaire has not been validated compared to a criterion measure. Reporting errors could introduce some bias in our results, particularly for the risk estimates for changing to an adjacent duration category. However, reporting errors are expected to attenuate the associations observed. For MVPA the recall period for the Time 1 (past 10 years) questionnaire was different than for Time 2 (past year), but our results for MVPA were similar in magnitude and direction to previous studies that have examined changes in MVPA and mortality risk among older adults [3, 4, 31] providing some reassurance that our harmonization efforts for MVPA were reasonable. Although we included those with preexisting conditions to increase both our sample-size and generalizability, our results are most applicable to older adults who are likely to live into their early 70’s with similar behavioral, demographic and health characteristics.

## Conclusions

In summary, our data provide further evidence that prolonged television viewing is associated with greater mortality risk and we demonstrate that older adults who chose to reduce their television viewing time had lower mortality compared to those who consistently watched more television. Collectively, our data provide clinicians and public health professionals with new evidence to support behavioral interventions designed to reduce sedentary television viewing in favor of more physically active pursuits, preferably more MVPA. Future studies are needed to confirm our findings for changes in television and mortality and experimental trials are need to better understand the behavioral and biological mechanisms that mediate the associations observed in this report.

## Additional file

Additional file 1:
**Supplementnal Methods and Results.** (DOC 692 kb)
