# Supplementary material for: Impact of changes in television viewing time and physical activity on longevity: a prospective cohort study
Source: Int J Behav Nutr Phys Act. 2015 Dec 18;12:156. doi: 10.1186/s12966-015-0315-0 (PMC4683741; doi:10.1186/s12966-015-0315-0)
Supplement: Additional file 1: — Supplementnal Methods and Results. (DOC 692 kb) [file 12966_2015_315_MOESM1_ESM.doc]

**Appendix: Assessment of television viewing and moderate-to-vigorous physical activity (MVPA) participation**

Television viewing

At Time 1, television viewing was assessed by the following question.

At Time 2, participants were asked a similar question.

We harmonized the television responses into the most meaningful categories allowed by both instruments, and that also ensured adequate cell sizes for our change analyses. Specifically, we classified responses from both time-points into <3, 3-4, and 5+ h/day categories.

|  |  |
| --- | --- |

Moderate-to-Vigorous Physical Activity

Physical activity was measured at Time 1 by asking about the average time spent each week in the past 10 years in activities of a moderate-vigorous intensity using the following question. The example activities provided in the question were primarily leisure-time/exercise and sports activities.

At Time 2, participants were asked about 16 different activities and the eight activities indicated below were used to estimate participation in MVPA.

For our change analysis, we harmonized the physical activity data to reflect MVPA participation at both time points and then categorized the duration responses into those with public health relevance as allowed by the questionnaires, while ensuring adequate numbers for the change analysis. Specifically, we classified MVPA into <1, 1-4, and >4 h/wk categories.

**Additional Table 1. Associations between television viewing and MVPA participation on mortality for those who reported the same behavior at both-time points, overall and stratified by follow-up time (< 3, ≥ 3 yrs): the NIH-AARP Diet and Health Study.**

|  |  | **Follow-up time** | |
| --- | --- | --- | --- |
| **No. deaths** | **Overall**  20,104 | **<3 yrs**  6,587 | **≥3yrs**  13,517 |
| **Television viewing** | | | |
| **<3 h/day** | 1.0 (referent) | 1.0 (referent) | 1.0 (referent) |
| **3-4h/day** | 1.13 (1.08, 1.18) | 1.11 (1.02, 1.20) | 1.13 (1.07, 1.19) |
| **5+h/day** | 1.28 (1.21, 1.34) | 1.33 (1.22, 1.45) | 1.24 (1.17, 1.32) |
| **MVPA** | | | |
| **<1h/wk** | 1.0 (referent) | 1.0 (referent) | 1.0 (referent) |
| **1-4h/wk** | 0.75 (0.71, 0.80) | 0.69 (0.62, 0.76) | 0.79 (0.73, 0.85) |
| **4+h/wk** | 0.66 (0.63, 0.70) | 0.60 (0.55, 0.65) | 0.71 (0.68, 0.75) |

* Values are adjusted* HR and 95% Cis. Participants in this analysis reported the same category of television viewing or MVPA at both Time 1 (1995-1996) and Time 2 (2004-2006). Fully adjusted model: age (yrs), sex (male or female), race (white, black, other, or missing), education (<12 yrs, high school graduate, some college, college graduate, or missing), smoking history (never; quit, <20 cigarettes/d; quit, >20 cigarettes/d; current, <20 cigarettes/d; current, .20 cigarettes/d; or unknown), history of heart disease (yes/no, missing), other chronic conditions (yes/no, missing), depression (yes/no, missing) health status (fair, good, very good or excellent), and BMI (18.5 to <25, 25-<30, 30-35, >35 kg/m2, or missing) and MVPA or television viewing respectively.

**Additional Table 2. Associations between changes in television viewing and MVPA participation stratified by follow-up time (< 3, ≥3 yrs)**

1. Television Viewing

|  |  |  | **Follow-up time** | |
| --- | --- | --- | --- | --- |
|  |  | **Overall** | **<3 yrs** | **≥ 3 yrs** |
| **No. Deaths** | | 20,104 | 6,587 | 13,517 |
| **Time 1** | **Time 2** |  |  |  |
| **<3 h/d** | **<3 h/d** | 1.0 (referent) | 1.0 (referent) | 1.0 (referent) |
|  | **3-4h/d** | 1.17 (1.10, 1.24) | 1.28 (1.15, 1.41) | 1.14 (1.05, 1.21) |
|  | **5+h/d** | 1.45 (1.32, 1.58) | 1.56 (1.34, 1.81) | 1.39 (1.25, 1.56) |
| **3-4 h/d** | **<3 h/d** | 0.91 (0.87, 0.96) | 0.93 (0.85, 1.02) | 0.90 (0.85, 0.96) |
|  | **3-4h/d** | 1.0 (referent) | 1.0 (referent) | 1.0 (referent) |
|  | **5+h/d** | 1.14 (1.09, 1.20) | 1.25 (1.15, 1.37) | 1.09 (1.03, 1.16) |
| **5+ h/d** | **<3 h/d** | 0.88 (0.79, 0.97) | 0.94 (0.79, 1.11) | 0.85 (0.74, 0.96) |
|  | **3-4h/d** | 0.85 (0.80, 0.91) | 0.87 (0.78, 0.97) | 0.84 (0.78, 0.91) |
|  | **5+h/d** | 1.0 (referent) | 1.0 (referent) | - 1. (referent) |

| 1. **MVPA** | |  | **Follow-up time** | |
| --- | --- | --- | --- | --- |
|  |  | **Overall** | **<3 yrs** | **≥ 3yrs** |
|  |  |  |  |  |
|  | **No. Deaths** | 20,104 | 6,587 | 13,517 |
| **Time 1** | **Time 2** |  |  |  |
|  | **<1 h/wk** | 1.0 (referent) | 1.0 (referent) | 1.0 (referent) |
| **<1 h/wk** | **1-4h/wk** | 0.82 (0.77, 0.87) | 0.79 (0.72, 0.88) | 0.84 (0.78, 0.90) |
|  | **4+h/wk** | 0.67 (0.62, 0.72) | 0.60 (0.53, 0.68) | 0.70 (0.64, 0.77) |
|  | **<1 h/wk** | 1.29 (1.21, 1.38) | 1.42 (1.27, 1.59) | 1.22 (1.13, 1.33) |
| **1-4h/wk** | **1-4h/wk** | 1.0 (referent) | 1.0 (referent) | 1.0 (referent) |
|  | **4+h/wk** | 0.90 (0.84, 0.96) | 0.88 (0.78, 1.0) | 0.90 (0.83, 0.97) |
|  | **<1 h/wk** | 1.38 (1.30, 1.45) | 1.49 (1.36, 1.64) | 1.32 (1.24, 1.41) |
| **4+h/wk** | **1-4h/wk** | 1.21 (1.16, 1.27) | 1.26 (1.16, 1.37) | 1.19 (1.13, 1.26) |
|  | **4+h/wk** | 1.0 (referent) | 1.0 (referent) | 1. (referent) |

Note: Chronic conditions included reported history of cardiovascular disease, cancer, renal disease or neurodegenerative conditions (Parkinson’s Diease, Amyotrophic lateral sclerosis or Multiple Sclerosis).

Fully adjusted model: age (yrs), sex (male or female), race (white, black, other, or missing), education (<12 yrs, high school graduate, some college, college graduate, or missing), smoking history (never; quit, <20 cigarettes/d; quit, >20 cigarettes/d; current, <20 cigarettes/d; current, .20 cigarettes/d; or unknown), history of heart disease (yes/no, missing), other chronic conditions (yes/no , missing), depression (yes/no, missing), health status (fair, good, very good or excellent), and change in BMI category from Time 1 to Time 2 (<25, 25-<30, >30, or missing) and television viewing.

**Additional Table 3. Participant Characteristics at Time 2 by Television Viewing Change Categories**

|  |  | | | | | | | | |
| --- | --- | --- | --- | --- | --- | --- | --- | --- | --- |
| **Time 1** | **< 3 h/day** | | | **3-4h/day** | | | **5+ h/day** | | |
| **Time 2** | **<3 h/day** | **3-4 h/day** | **5+h/day** | **<3 h/day** | **3-4 h/day** | **5+h/day** | **<3 h/day** | **3-4 h/day** | **5+h/day** |
| N | 45153 | 13497 | 3796 | 22599 | 34911 | 14959 | 3211 | 10754 | 16307 |
| Age (y) | 69.8 (5.4) | 70.3 (5.2) | 70.2 (5.2) | 70.2 (5.2) | 70.7 (5.4) | 71.2 (5.2) | 71.1 (5.1) | 70.9 (5.3) | 71.5 (5.2) |
| BMI | 25.7 (4.1) | 26.7 (4.4) | 27.2 (4.8) | 27.2 (4.8) | 26.6 (4.4) | 26.9 (4.4) | 27.7 (5.0) | 27.3 (4.9) | 28.4 (5.5) |
| Education (some college) | 79.5 | 72.5 | 67.2 | 68.0 | 65.3 | 60.0 | 55.9 | 55.6 | 52.7 |
| Married | 71.9 | 69.4 | 66.0 | 70.9 | 71.2 | 66.4 | 65.0 | 66.6 | 60.6 |
| Race (white) | 94.7 | 94.4 | 93.1 | 93.4 | 94.3 | 92.8 | 89.4 | 91.8 | 91.1 |
| Very good/excellent health | 64.4 | 53.2 | 47.7 | 55.4 | 49.8 | 41.9 | 48.1 | 42.8 | 36.0 |
| Depression | 11.5 | 13.1 | 16.0 | 11.5 | 11.5 | 14.6 | 15.1 | 13.5 | 16.0 |
| Sleep < 7 h/night | 24.4 | 24.0 | 26.5 | 30.2 | 27.7 | 27.4 | 38.1 | 33.7 | 31.3 |
| Coronary heart disease | 16.7 | 21.3 | 24.0 | 20.1 | 22.6 | 25.2 | 22.9 | 24.7 | 26.4 |
| Cancer | 12.7 | 13.3 | 13.4 | 12.9 | 13.7 | 14.2 | 12.8 | 13.8 | 14.2 |
| Chronic condition | 5.2 | 7.3 | 9.8 | 7.0 | 7.9 | 10.5 | 10.1 | 9.7 | 12.3 |
| Degenerative disease | 0.7 | 1.2 | 1.2 | 0.7 | 0.9 | 1.2 | 1.0 | 0.9 | 1.2 |

Information at Time 2 was reported on the Follow-up Questionnaire, administered 2004-2006. Values in parentheses are standard deviation. Degenerative diseases includes Parkinson’s Disease, Amyotrophic lateral sclerosis or Multiple Sclerosis.

**Table 4. Participant Characteristics at Time 2 by Changes in Moderate to Vigorous Physical Activity (MVPA)**

| **Time 1** | **<1 h/wk** | | | **1-4 h/wk** | | | **4+ h/wk** | | |
| --- | --- | --- | --- | --- | --- | --- | --- | --- | --- |
| **Time 2** | **<1 h/wk** | **1-4 h/wk** | **4+h/wk** | **<1 h/wk** | **1-4 h/wk** | **4+h/wk** | **<1 h/wk** | **1-4 h/wk** | **4+h/wk** |
| N | 14403 | 13927 | 9355 | 10421 | 16362 | 15483 | 13234 | 23889 | 48113 |
| Age (y) | 70.4 (5.4) | 70.4 (5.4) | 70.1 (5.3) | 70.1 (5.3) | 70.5 (5.5) | 70.6 (5.4) | 70.2 (5.2) | 71.2 (5.3) | 71.2 (5.3) |
| BMI | 28.9 (5.8) | 27.9 (5.2) | 27.4 (4.7) | 27.4 (4.7) | 27.9 (5.2) | 27.0 (4.6) | 26.7 (4.2) | 27.1 (4.8) | 25.7 (3.7) |
| Education (some college) | 60.6 | 65.1 | 69.5 | 61.5 | 69.3 | 74.9 | 54.6 | 64.0 | 73.9 |
| Married | 63.5 | 65.3 | 70.3 | 65.3 | 67.5 | 73.2 | 65.3 | 67.6 | 73.8 |
| Race (white) | 91.5 | 92.1 | 92.0 | 92.8 | 93.8 | 93.8 | 93.4 | 94.1 | 94.7 |
| Very good/excellent health | 34.2 | 41.9 | 50.3 | 39.7 | 48.9 | 58.9 | 42.0 | 51.3 | 65.8 |
| Depression | 16.8 | 14.8 | 12.2 | 15.8 | 13.5 | 10.8 | 15.5 | 13.0 | 9.4 |
| Sleep < 7 h/night | 31.8 | 29.0 | 26.9 | 29.9 | 27.4 | 25.5 | 31.8 | 27.8 | 25.5 |
| Coronary heart disease | 23.2 | 22.4 | 23.9 | 21.5 | 20.5 | 21.3 | 22.2 | 20.9 | 20.2 |
| Cancer | 13.0 | 12.8 | 13.2 | 14.0 | 12.9 | 12.9 | 14.0 | 13.8 | 13.4 |
| Chronic condition | 11.8 | 9.4 | 8.7 | 10.5 | 7.6 | 6.3 | 10.7 | 7.9 | 5.4 |
| Degenerative disease | 1.2 | 1.0 | 0.9 | 1.3 | 1.0 | 0.8 | 1.4 | 0.9 | 0.6 |

Information at Time 2 was reported on the Follow-up Questionnaire, administered 2004-2006. Values in parentheses are standard deviation. Degenerative diseases includes Parkinson’s Disease, Amyotrophic lateral sclerosis or Multiple Sclerosis.

**Additional Table 5. Sensitivity analysis for long-term mortality associations excluding first year of follow-up and those reporting chronic conditions at Time 2 (2004-2006).**

1. Long-term television viewing (same category reported at both Time 1 and Time 2)

|  | **Overall** | **>1 yr**  **follow-up** | **Excluding**  **chronic conditions** |
| --- | --- | --- | --- |
| **No. Deaths** | 20,387 | 18,506 | 8,231 |
| **<3 h/d** | 1.0 (referent) | 1.0 (referent) | 1.0 (referent) |
| **3-4h/d** | 1.13 (1.08, 1.18) | 1.14 (1.09, 1.19) | 1.12 (1.04, 1.18) |
| **5+h/d** | 1.28 (1.21, 1.34) | 1.29 (1.22, 1.35) | 1.31 (1.21, 1.41) |

1. Long-term MVPA (same category reported at both Time 1 and Time 2)

|  |  | **Overall** | **>1 yr follow-up** | **Excluding chronic conditions** |
| --- | --- | --- | --- | --- |
|  | **No. Deaths** | 20,104 | 18,758 | 8,231 |
| **Time 1**  **1995-1996** | **Time 2**  **2004-2005** |  |  |  |
| **<1h/wk** | **<1h/wk** | 1.0 (referent) | 1.0 (referent) | 1.0 (referent) |
| **1-4h/wk** | **1-4h/wk** | 0.75 (0.71, 0.80) | 0.77 (0.72, 0.82) | 0.71 (0.65, 0.78) |
| **4+h/wk** | **4+h/wk** | 0.66 (0.63, 0.70) | 0.68 (0.64, 0.72) | 0.67 (0.62,0.73) |

Note: Chronic conditions included reported history of cardiovascular disease, cancer, renal disease or neurodegenerative conditions (Parkinson’s Diease, Amyotrophic lateral sclerosis or Multiple Sclerosis).

Fully adjusted model: age (yrs), sex (male or female), race (white, black, other, or missing), education (<12 yrs, high school graduate, some college, college graduate, or missing), smoking history (never; quit, <20 cigarettes/d; quit, >20 cigarettes/d; current, <20 cigarettes/d; current, .20 cigarettes/d; or unknown), history of heart disease (yes/no, missing), other chronic conditions (yes/no , missing), depression (yes/no, missing), health status (fair, good, very good or excellent), and change in BMI category from Time 1 to Time 2 (<25, 25-<30, >30, or missing) and television viewing.

**Additional Table 6. Sensitivity Analyses for association between changes in MVPA and mortality: the NIH-AARP Diet and Health Study**

| **Time 1** |  | **<3 h/day** | | | **3-4 h/day** | | | **5+ h/day** | | |
| --- | --- | --- | --- | --- | --- | --- | --- | --- | --- | --- |
| **Time 2** | No. deaths | **<3 h/d** | **3-4h/d** | **5+h/d** | **<3 h/d** | **3-4h/d** | **5+h/d** | **<3 h/d** | **3-4h/d** | **5+h/d** |
| **Overall** | 20,104 | **1.0** (referent) | **0.82** (0.77,0.87) | **0.67** (0.62,0.72) | **1.29** (1.21,1.38) | **1.0** (referent) | **0.90** (0.84,0.96) | **1.38** (1.30,1.45) | **1.21** (1.16,1.27) | **1.0** (referent) |
| **Men** | 13,251 | **1.0** (referent) | **0.84** (0.78,0.91) | **0.67** (0.61,0.73) | **1.34** (1.23,1.45) | **1.0** (referent) | **0.92** (0.85,1.00) | **1.37** (1.28,1.47) | **1.22** (1.15,1.29) | **1.0** (referent) |
| **Women** | 6,853 | **1.0** (referent) | **0.80** (0.72,0.88) | **0.67** (0.59,0.77) | **1.21** (1.08,1.35) | **1.0** (referent) | **0.84** (0.74,0.95) | **1.39** (1.26,1.52) | **1.19** (1.10,1.30) | **1.0** (referent) |
| **Normal weight** | 8,560 | **1.0** (referent) | **0.79** (0.71,0.87) | **0.64** (0.57,0.72) | **1.35** (1.22,1.50) | **1.0** (referent) | **0.85** (0.76,0.94) | **1.34** (1.24,1.46) | **1.19** (1.11,1.28) | **1.0** (referent) |
| **Overweight/obese** | 11,544 | **1.0** (referent) | **0.85** (0.78,0.91) | **0.68** (0.62,0.75) | **1.26** (1.16,1.37) | **1.0** (referent) | **0.93** (0.85,1.02) | **1.40** (1.30,1.51) | **1.22** (1.14,1.30) | **1.0** (referent) |
| **Younger** | 5,968 | **1.0** (referent) | **0.93** (0.84,1.04) | **0.76** (0.67,0.86) | **1.32** (1.16,1.49) | **1.0** (referent) | **0.87** (0.76,0.99) | **1.20** (1.07,1.33) | **1.18** (1.08,1.29) | **1.0** (referent) |
| **Older** | 8,231 | **1.0** (referent) | **0.78** (0.72,0.84) | **0.62** (0.57,0.68) | **1.28** (1.18,1.38) | **1.0** (referent) | **0.91** (0.83,0.98) | **1.45** (1.36,1.55) | **1.22** (1.16,1.29) | **1.0** (referent) |
| **> 1 yr Follow-up** | 18,506 | **1.0** (referent) | **0.83** (0.78,0.89) | **0.69** (0.64,0.75) | **1.27** (1.18,1.36) | **1.0** (referent) | **0.89** (0.83,0.95) | **1.37** (1.29,1.45) | **1.21** (1.15,1.27) | **1.0** (referent) |
| **Excluding chronic conditions** | 8,231 | **1.0** (referent) | **0.82** (0.75,0.90) | **0.71** (0.63,0.80) | **1.38** (1.24,1.53) | **1.0** (referent) | **0.94**  (0.84,1.04) | **1.34** (1.23,1.46) | **1.18** (1.10,1.27) | **1.0** (referent) |

Note: Chronic conditions included reported history of cardiovascular disease, cancer, renal disease or neurodegenerative conditions (Parkinson’s Diease, Amyotrophic lateral sclerosis or Multiple Sclerosis). Younger individuals were below median age (71.2y at Time 2) and older were greater than or equal to the median age. Nomal-weight was BMI <25 kg/m^2^ and overweight/obese was greater than or equal to 25 kg/m^2^.

Fully adjusted model: age (yrs), sex (male or female), race (white, black, other, or missing), education (<12 yrs, high school graduate, some college, college graduate, or missing), smoking history (never; quit, <20 cigarettes/d; quit, >20 cigarettes/d; current, <20 cigarettes/d; current, .20 cigarettes/d; or unknown), history of heart disease (yes/no, missing), other chronic conditions (yes/no , missing), depression (yes/no, missing), health status (fair, good, very good or excellent), and change in BMI category from Time 1 to Time 2 (<25, 25-<30, >30, or missing) and television viewing.
